# Mirasol pathogen reduction technology treatment of human whole blood does not induce acute lung injury in mice

**DOI:** 10.1371/journal.pone.0178725

**Published:** 2017-06-01

**Authors:** Beñat Mallavia, Nicholas Kwaan, Susanne Marschner, Susan Yonemura, Mark R. Looney

**Affiliations:** 1 Department of Medicine, University of California San Francisco, San Francisco, California, United States of America; 2 Terumo BCT, Inc., Lakewood, Colorado, United States of America; 3 Department of Laboratory Medicine, University of California San Francisco, San Francisco, California, United States of America; Emory University/Georgia Institute of Technology, UNITED STATES

## Abstract

In resource-limited settings and in the military theater, fresh human whole blood is commonly transfused, but infectious risks are a concern. Sophisticated molecular testing for potential infectious agents in the whole blood is often unavailable. To address this unmet need, pathogen reduction technology (PRT) has been developed, and it is an effective approach to inactivate a broad range of pathogens found in human blood. However, studies are needed to determine if it is harmful to blood cells and whether these cells could damage the transfused recipient, including the development of acute lung injury/acute respiratory distress syndrome. In this study, we used a commercial PRT system to treat human whole blood that was then transfused into immunodeficient mice, and the development of acute lung injury was determined. In a model of transfusion-related acute lung injury (TRALI), BALB/c SCID mice developed more robust lung injury when challenged with a MHC Class I monoclonal antibody compared to BALB/c wild-type and NOD/SCID mice. Transfusion of control versus Mirasol PRT-treated whole blood (25% blood volume exchange) into BALB/c SCID mice did not produce lung injury at storage day 1. However, mild lung injury at storage days 14 and 21 was observed without significant differences in lung injury measurements between Mirasol PRT-treated and control groups. The mild storage-dependent acute lung injury correlated with trends for increased levels of cell-free hemoglobin that accumulated in both the control and Mirasol PRT-treated groups. Neutrophil extracellular traps were elevated in the plasma of BALB/c SCID mice in the monoclonal antibody TRALI model, but were not different in mice that received exchange transfusions. In conclusion, exchange transfusion of human whole blood into immunodeficient mice produces mild lung injury that is storage-dependent and not related to pathogen reduction treatment.

## Introduction

Although the safety of the blood supply has improved with the advent of modern transfusion therapy, a variety of pathogens, known and unknown, continue to provide risk to transfused recipients [1]. Current screening relies on donor questionnaires and limited laboratory testing for specific pathogens. Molecular testing for viral pathogens has been successful in reducing transmission risk [2], however, these tests are impractical when there is not enough time or resources to screen for pathogens, such as the use of fresh, warm whole blood in military settings [3–6]. Whole blood is also the most common product transfused in low-income countries [7].

A variety of pathogen-reduction technologies (PRT) have been developed to address the potential infectious complications of transfusion therapy [8]. While these technologies can effectively eliminate a variety of possible pathogens, damage to cells in the blood product may occur. A prior study found that patients transfused with psoralen-based PRT of human platelet units had increased cases of the acute respiratory distress syndrome (ARDS), which may have been related to PRT-induced platelet activation [9]. These cases of ARDS are akin to transfusion-related acute lung injury (TRALI), which is the leading cause of transfusion-related death in many countries [10]. TRALI most commonly results from the transfusion of cognate HLA antibody to “primed” recipients [11], and can be modeled in mice using a MHC Class I monoclonal antibody that produces neutrophil-, platelet-, and monocyte-dependent lung injury [12–16].

Here, we tested Mirasol^®^ PRT, a technology that utilizes ultraviolet light illumination of human blood to which a photosensitizing agent, (riboflavin, vitamin B_2_) has been added [17]. This process creates nucleic acid modifications, which effectively inactivates a variety of pathogens. Our prior study in apheresis platelets showed that Mirasol PRT produced platelet activation during storage, but did not lead to the development of lung injury [18]. In this study, we tested whether Mirasol-treated stored, human whole blood, when transfused into immunodeficient mice, produces lung injury. We also examined whether human whole blood treated with Mirasol PRT induces the development of neutrophil extracellular traps (NETs) [19] after exchange transfusion, since NETs are implicated in the pathogenesis of transfusion-related acute lung injury (TRALI) [20, 21].

## Materials and methods

### Mice

BALB/c, BALB/c SCID and NOD/SCID mice (Jackson Laboratories) at 8–10 weeks of age were used for all experiments and were housed in pathogen-free conditions.

### Ethics statement

Blood was obtained from Bonfils Blood Center (Denver, CO) and the authors had no contact with the blood donors. Thus, this study does not involve human subjects research. The study was conducted in strict accordance with the recommendations in the Guide for the Care and Use of Laboratory Animals of the National Institutes of Health. The Institutional Animal Care and Use Committee at the University of California, San Francisco (Protocol #AN099492) as well as by the United States Army Medical Research and Materiel Command (USAMRMC) Animal Care and Use Review Office (ACURO) (Protocol #12229090), approved this protocol. We used ketamine and xylazine anesthesia for all surgery and made all efforts to minimize suffering.

### Human blood collection and Mirasol PRT treatment

Human whole blood was collected under an IRB (Western Institutional Review Board, Protocol #20090968) and USAMRMC Human Research Protection Office (HRPO, Protocol A-17635.2)-approved research blood collection protocol at Bonfils Blood Center (Denver, CO) in routine 450 mL blood collection sets with citrate-phosphate-dextrose (CPD) as a preservative. The whole blood was shipped overnight at room temperature to the University of California, San Francisco, and Mirasol PRT treatment was done on storage day 1. Each unit of whole blood was transferred to a Mirasol Illumination Bag (MIB) while a control unit remained in the collection bag. Prior to treatment with the Mirasol system, thirty-five (35) mL of 500 μM riboflavin solution was added to the test units. The Mirasol illuminator provided a UV energy dose of 80 J/mL_RBC_. For untreated control units, 35 mL of normal saline was added to mimic the dilution of the test products with riboflavin. All products were stored under standard blood banking conditions at 4°C until the time of experimentation. To measure cell-free hemoglobin, 2 mL of blood was centrifuged at 2,000 g for 15 minutes and then the supernatant was centrifuged at 3,000 g for 10 minutes. The hemoglobin of the final supernatant was measured using a HemaCue^®^ Plasma/Low Hb System (HemaCue, Brea, CA).

### Mouse model and acute lung injury measurements

BALB/c SCID mice were primed with LPS (Escherichia coli O55:B5; Sigma-Aldrich, St. Louis, MO, dose 0.1 mg/kg, i.p., 24 hours prior). The mice were then anesthetized with ketamine and xylazine and an exchange transfusion was performed via a 30 gauge needle inserted into the jugular vein. The mouse blood volume (mL) was estimated by multiplying the body weight in grams x 7%. For example, a 25 gram mouse was estimated to have a blood volume of 1.75 mL. We then removed 25% of this volume (438 μL) from the jugular vein over 2–3 minutes followed immediately by transfusion of human whole blood of identical volume administered over 2–3 minutes. The following conditions were tested: 25% estimated whole blood exchange transfusion (untreated control vs. Mirasol PRT) on days 1, 14, and 21 of storage.

Mice were euthanized at 4 hours after transfusion consistent with the susceptible period of TRALI development [13], and we collected blood and lungs for measurements of acute lung injury. Extravascular lung water was measured by the gravimetric method as previously described [13]. We measured lung vascular permeability by injecting ^125^I-albumin (Iso-Tex Diagnostics, Friendswood, TX) in the jugular vein of the mice at the time of transfusion and then measuring the ratio of radioactivity between the blood and the bloodless lung using a gamma counter (Packard 5000 Series, Ramsey, MN) [13]. A Hemavet 950 machine (Drew Scientific, Miami Lakes, FL) was used to measure hemoglobin and hematocrit in the mouse samples.

### TRALI model

We used our previously described two-event model of TRALI [12]. Briefly, after priming with LPS (0.1 mg/kg, i.p.) 24 hours prior, mice were administered a cognate MHC Class I monoclonal Ab (mAb) (ATCC 34-1-2S; H-2K^d^; IgG_2a_, κ; 1.0 mg/kg) by jugular vein injection and euthanized at 4 hours. In selected experiments, mice were challenged with MHC Class I mAb alone (no LPS priming). Blood and lungs were collected to measure extravascular lung water and lung vascular permeability. Plasma was collected in all experiments for NET analysis. In a subset of experiments, lungs were collected in 4% paraformaldehyde or OCT for histological analysis with hematoxylin and eosin staining, as previously described [12].

### NETs ELISA

To determine the presence of neutrophil extracellular traps (NETs), we measured neutrophil elastase (NE)–DNA complexes using a sandwich ELISA, as previously described [20, 22]. Briefly, 96-well microtiter plates were incubated with anti-mouse NE antibody (sc-9521, Santa Cruz Biotechnology, Inc., Dallas, TX) overnight at 4°C. After washing wells with PBS and blocking with 5% bovine serum albumin (BSA), 50 μL of plasma was added to wells in triplicate and incubated for 2 hours at room temperature on an orbital shaker. Wells were then washed again and incubated with 50 μL of peroxidase-conjugated anti-DNA antibody (Cell Death Detection ELISA Kit, Roche Applied Science, Indianapolis, IN) diluted 1:100 in incubation buffer for 2 hours at room temperature. After thorough washing, 100 μL of 1-Step ABTS reagent (Thermo Scientific, Waltham, MA) was added to each well and the plate incubated for 30 minutes at room temperature. The optical densities (OD) at 405nm and 490nm wavelengths were measured by plate reader and the difference between the two (OD405-490) was then applied to a standard curve derived from bronchoalveolar lavage samples obtained from mice with acute lung injury to yield NET arbitrary units [22].

### Statistical analysis

Results are reported as mean ± SEM. We used 2-tailed Student’s t and ANOVA tests to determine significance, as appropriate (GraphPad PRISM version 6.0). Log-rank testing was used for survival analysis. *P* values of less than or equal to 0.05 were considered to be significant.

## Results

### Mirasol treatment of whole blood does not increase extracellular hemoglobin

**Fig 1:**
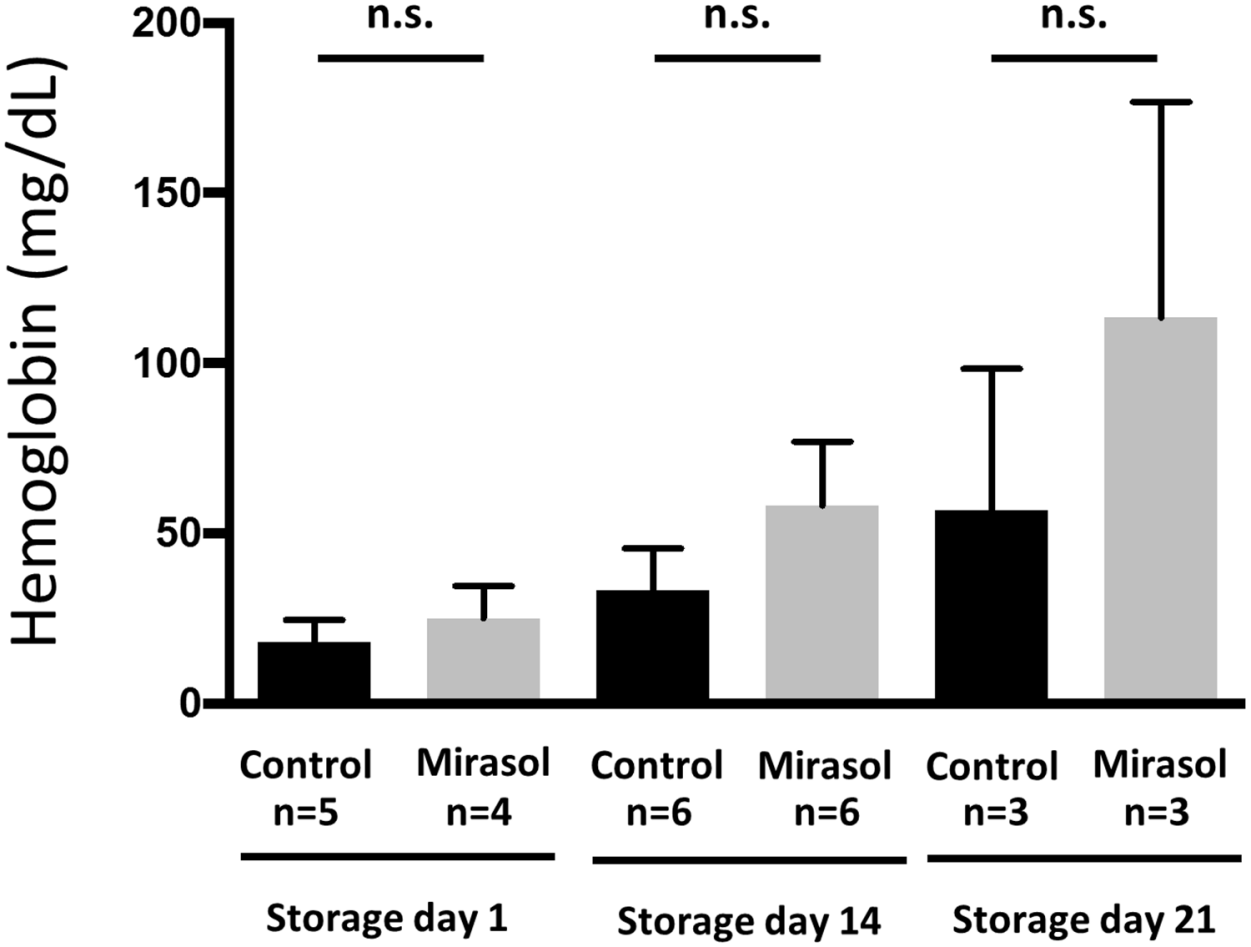
Cell-free hemoglobin measured in control and Mirasol treated whole blood. Mirasol PRT treatment did not significantly change the amount of cell-free hemoglobin measured in supernatants of whole blood bags. Between group measurements are not statistically different at 1, 14, and 21 days of blood storage. Mean ± SEM. n = 3–6 per group. p = 0.19 for trend in hemoglobin values in control group (ANOVA). p = 0.08 for trend in hemoglobin values in Mirasol group (ANOVA).

We measured cell-free hemoglobin in human whole blood units on storage days 1, 14 and 21. At each time point, we compared untreated control and Mirasol PRT-treated units. Hemoglobin values were not significantly different between the groups (Fig 1). There was a non-significant overall trend for increased cell-free hemoglobin with increased storage days.

### BALB/c SCID mice have increased lung injury and decreased survival in TRALI

**Fig 2:**
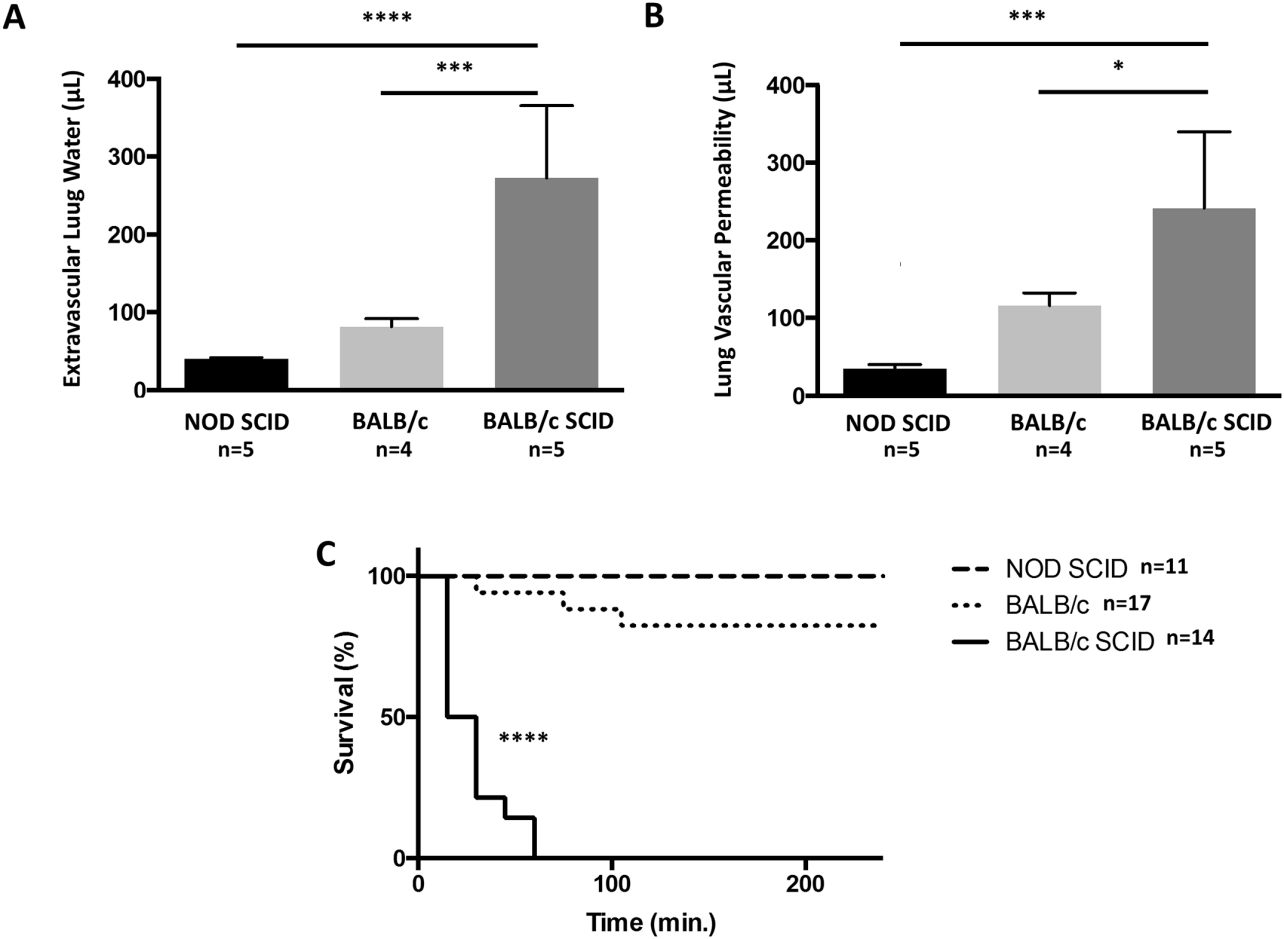
Lung injury and survival in mice with TRALI. LPS-primed mice challenged with MHC Class I mAb (H-2^d^) produced increased lung injury measured by (**A**) extravascular lung water and (**B**) lung vascular permeability in BALB/c SCID compared to NOD SCID mice. BALB/c wild-type mice also showed increased lung injury compared to NOD SCID mice, but significantly lower than their BALB/c SCID counterparts. Mean ± SEM. n = 4–5 per group. ****p<0.0001, ***p<0.001, *p<0.05 (ANOVA). (**C**) BALB/c SCID (n = 14) also showed decreased survival compared to NOD SCID (n = 11) and BALB/c wild-type (n = 17) groups. Mean ± SEM. ****p<0.0001 vs. NOD SCID and BALB/c wild-type (Log-rank test). PRT-treated human whole blood (pooled storage days). Mean ± SEM. *p<0.05 (ANOVA).

We used an established two-event TRALI model to challenge NOD SCID, BALB/c wild-type, and BALB/c SCID mice. BALB/c SCID mice had increased lung injury as measured by extravascular lung water and lung vascular permeability (2 to 3 fold higher) compared to NOD SCID mice or BALB/c wild-type mice (Fig 2A and 2B). BALB/c wild-type mice had a non-significant increase in lung injury compared to the NOD SCID mice and a small decrease in survival (Fig 2A–2C). Consistent with the severe lung injury measurements in BALB/c SCID mice, all of the mice died by 1 hour after antibody-challenge (Fig 2C). Since an immunodeficient mouse strain is required for the human whole blood transfusions, we concluded that BALB/c SCID mice are capable of producing robust lung injury and were the best choice for these experiments.

### Mirasol PRT-treated human whole blood does not produce increased lung injury compared to untreated control blood

**Fig 3:**
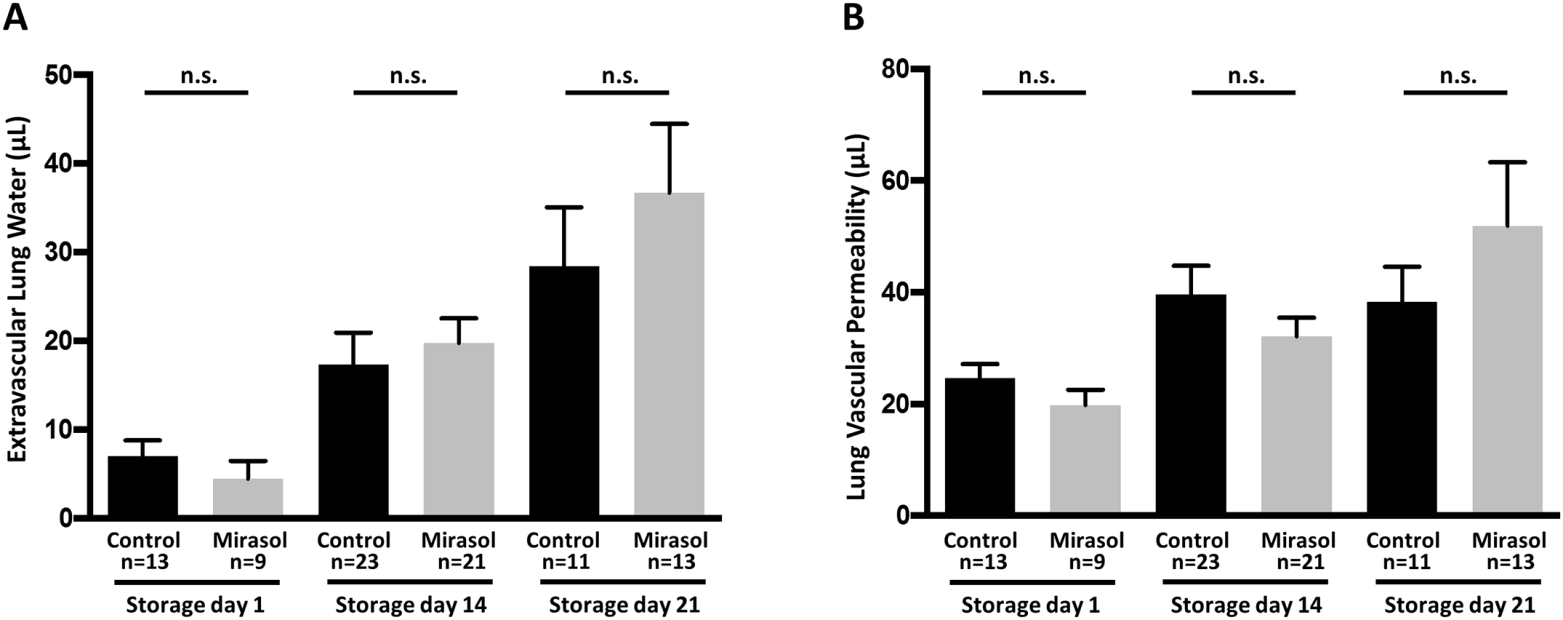
Lung injury measurements in control or Mirasol treated blood transfused into BALB/c SCID mice. LPS priming plus a 25% exchange transfusion using Mirasol PRT-treated human whole blood stored for 1 (n = 9), 14 (n = 21), or 21 (n = 13) days versus untreated control human whole blood stored for 1 (n = 13), 14 (n = 23), or 21 (n = 11) days did not generate significant differences in extravascular lung water (**A**) or lung vascular permeability (**B**) between the two groups (all storage times non-significant control vs. Mirasol). However, extravascular lung water and lung vascular permeability increased with blood storage time independent of Mirasol PRT treatment. For extravascular lung water, p<0.01 for trend in both Mirasol and control groups. For lung vascular permeability, p<0.01 for trend in Mirasol group and p = 0.11 for trend in control group (ANOVA). Mean ± SEM.

Human whole blood units were treated with the Mirasol PRT System or left untreated (control) and stored for 1, 14 or 21 days at 4°C. A 25% exchange transfusion in LPS-primed BALB/c SCID mice with Mirasol PRT-treated blood did not increase extravascular lung water (Fig 3A) or lung vascular permeability (Fig 3B) compared to exchange transfusion of untreated control blood at any of the storage time points. However, in both groups, there was a statistically significant trend for increased mild lung injury with longer storage times (ANOVA post-test for linear trend).

**Fig 4:**
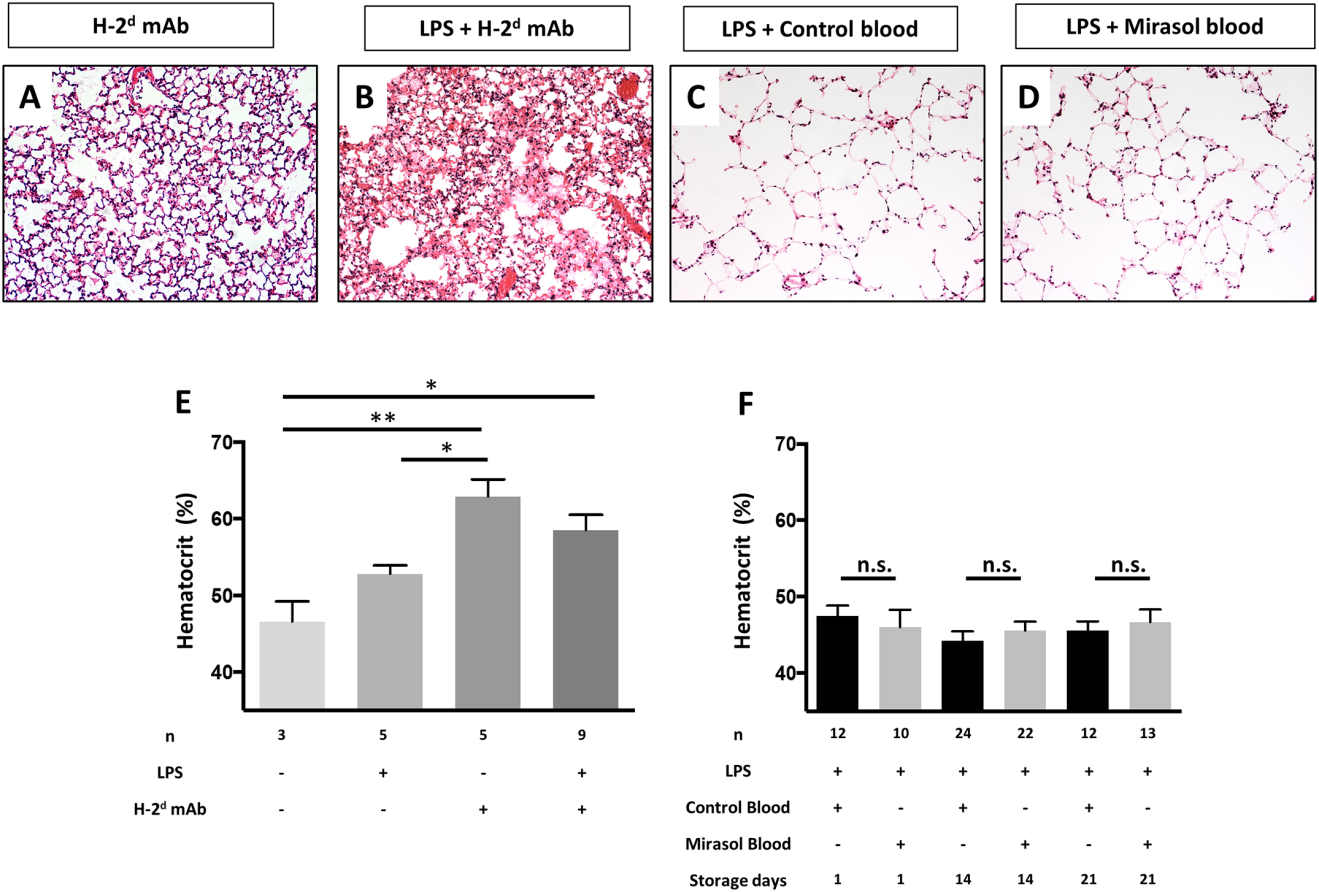
Representative lung histology and hematocrit values. H&E staining on lung sections from (**A**) BALB/c SCID mice + H-2^d^ mAb, (**B**) BALB/c SCID mice + LPS priming + H-2^d^ mAb, (**C**) BALB/c SCID mice + LPS priming and a 25% exchange transfusion using control human whole blood (storage day 21), and (**D**) BALB/c SCID mice + LPS priming and a 25% exchange transfusion using Mirasol-PRT treated human whole blood (storage day 21). Extensive lung injury (interstitial and alveolar edema, leukocyte infiltration) is observed in the TRALI groups (**A**, **B**), but no lung injury is detectable in either group of mice transfused with human whole blood (**C, D**). 20x magnification. (**E**) Hematocrit values in blood samples obtained from controls, TRALI, and (**F**) human whole blood challenged animals. Hemoconcentration is observed with TRALI, but not in the other groups. Mean ± SEM. ****p<0.0001, **p<0.01 (ANOVA).

BALB/c SCID mice challenged with either H-2^d^ mAb alone or two-event TRALI had severe histological lung injury with leukocyte infiltration and interstitial and alveolar edema present (Fig 4A and 4B). In mice challenged with LPS priming plus either control or Mirasol PRT-treated whole blood, there were no differences in histological lung injury on any of the storage days. There is minimal to no evidence of histological lung injury in mice challenged with whole blood on storage day 21 (Fig 4C and 4D), which is consistent with the small increases in lung water and lung vascular permeability (Fig 3). As a measurement of systemic vascular leakage, BALB/c SCID mice demonstrated significant hemoconcentration with either H-2^d^ mAb alone or two-event TRALI (Fig 4E). LPS primed BALB/c SCID mice treated with an exchange transfusion with either control or Mirasol-treated human whole blood had normal hematocrits at all storage times (Fig 4F).

### Neutrophil extracellular trap release is increased in TRALI

**Fig 5:**
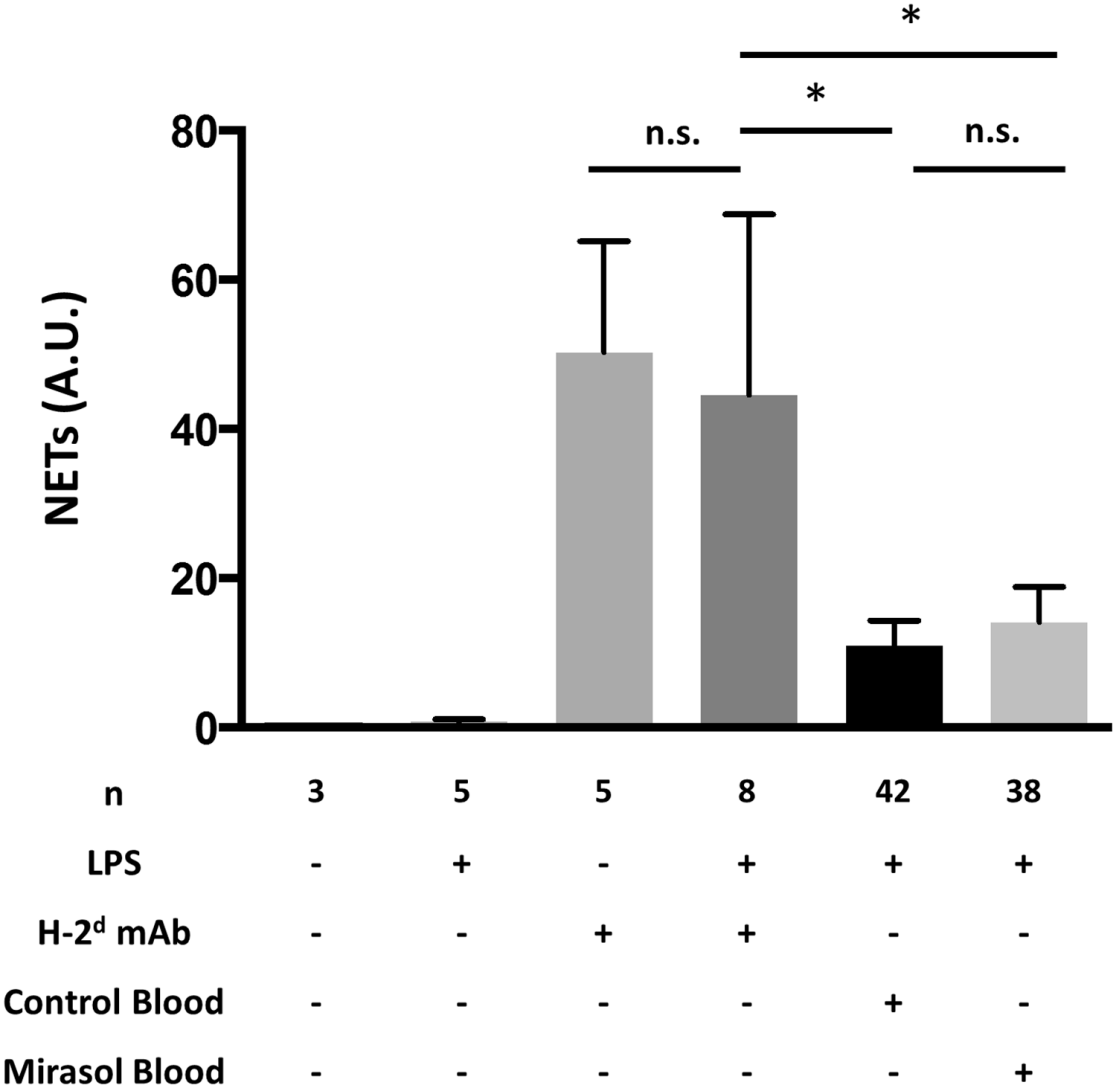
Presence of NETs in plasma after TRALI or human whole blood transfusion. Plasma NETs in BALB/c SCID mice challenged with no intervention, LPS priming, H-2^d^ mAb alone, LPS + H-2^d^ mAb, or LPS priming and either control or Mirasol.

Neutrophil activation and neutrophil extracellular trap (NET) release is an important inducer of acute lung injury. We have previously shown in BALB/c wild-type mice challenged with TRALI that NETs are increased in the blood compartment [20]. Here, we measured plasma NETs (by NE-DNA ELISA) in BALB/c SCID mice challenge with TRALI (± LPS priming) and in mice challenged with LPS + either control or Mirasol-treated human whole blood. NETs were increased in these groups, but significantly higher in the TRALI mice (Fig 5). There was no difference in plasma NETs between the control and Mirasol-treated human whole blood groups.

## Discussion

In this experimental transfusion study, our major findings are (1) BALB/c SCID mice are hyper-susceptible to a two-event model of TRALI and therefore a suitable immunodeficient mouse strain to test human blood products, (2) there is no differential effect of either control or Mirasol-treated human whole blood on lung injury, and (3) there is a mild increase in lung injury in both groups with longer durations of whole blood storage.

Blood-borne pathogens constitute an important risk for blood transfusions worldwide. Whereas in developed countries pathogen screening has reduced transfusion risks, there remain challenges in the developing world and resource limited settings where testing infrastructure may not be present. Another challenging setting for pathogen screening is within the military where the immediate need for transfusions in trauma in forward operating settings and lack of component blood supply necessitates the use of unscreened whole blood. Pathogen-reduction technology offers an attractive method to reduce transfusion-related infection risks. Several studies have shown that this technology can reduce the risk of pathogen transmission due to contaminated blood transfusions [23, 24].

Initial studies have shown that platelet concentrate units can be treated with riboflavin and UV light to reduce viral infections and bacteria counts without inducing acute lung injury [18, 24–26]. Although platelet activation by Mirasol treatment has been observed, the biological effect of this activation did not lead to acute lung injury [18, 27]. However, a recent work suggests that platelet transfusion clinical effectiveness might be reduced due to decreased normal platelet function after PRT procedures [28].

We focused on Mirasol PRT-treated whole blood and tested the possible generation of lung injury by transfusion of this treated blood. As a generalized indicator of whole blood quality, we measured free hemoglobin values in treated and untreated blood bags after storage. Our results revealed that Mirasol PRT did not induce more hemolysis compared to untreated control samples.

We utilized mice that were susceptible to lung injury in a well-established model of TRALI. Previously, we used NOD SCID immunodeficient mice that, despite lacking T or B cells and having impaired myeloid cell functions, develop moderate levels of lung injury in the TRALI model. BALB/c SCID mice share the severe immunodeficiency mutation with NOD SCID mice, which reduces humoral and cellular immunity, but BALB/c SCID mice have normal myeloid lineages, including neutrophils, which are key mediators of lung injury in TRALI. Mice with the same SCID mutation have previously been shown to be more sensitive to lung injury than their wild-type counterparts [29] due to the lack of CD4+ regulatory T cells and dendritic cells [30]. BALB/c SCID mice showed robust lung edema and permeability compared to either BALB/c wild-type or NOD SCID mice, which makes this model ideal to test possible TRALI induced by human blood transfusion.

In this work, we studied the safety of Mirasol PRT treatments applied to whole blood units and their relation to acute lung injury. The use of Mirasol treatment in human whole blood before transfusing it into BALB/c SCID mice did not increase lung edema or lung permeability compared with the untreated control blood. Our study found that blood storage time affects lung injury parameters in a Mirasol PRT treatment-independent fashion. Longer storage time showed a significant trend for increased extravascular lung water and lung vascular permeability. However, no histologic differences were noted, nor was there evidence of significant hemoconcentration suggestive of systemic vascular leak. Finally, we observed that blood treatment before transfusion did not differentially affect NET production. These data indicate that although there are measurable increases in lung injury with longer storage times using sensitive techniques, these effects may be subclinical.

Clinical outcome studies examining the effects of storage lesion, particularly in packed red blood cells, have produced conflicting results. A meta-analysis in 2012 suggested that use of older stored blood was associated with a 16% increase in risk of death [31]. However, three recent randomized controlled trials showed no difference between short-term storage versus longer storage of transfused blood in 90-day mortality for critically ill patients, in-hospital mortality for hospitalized patients, or Multiple Organ Dysfunction Score for those patients undergoing cardiac surgery [32–34]. Our study found a mild increase in lung injury with prolonged storage of human whole blood, but it would be very rare for whole blood to ever be transfused at >2 weeks of storage time [35].

Our work has limitations. Our 4-hour endpoint might not be sufficient time to observe longer-term effects induced by whole blood transfusion. However, TRALI is clinically defined as acute lung injury within 6 hours of transfusion, and most cases occur within 1–2 hours after transfusion [36]. Therefore, we believe that a 4-hour period of observation is sufficient. Also, transfusions using unscreened whole blood are rare in developed settings and limited to very specific conditions; this fact limits the scope of our study. Nevertheless, resource-limited settings and critical situations, such as in the military, require a readily available blood supply from healthy donors. Thus, we consider that this work remains relevant for certain clinical situations.

As a final conclusion, we demonstrated that although stored whole blood storage might induce mild lung injury, these effects are independent of PRT treatment. Our work demonstrates Mirasol PRT treatment does not independently induce lung injury in a murine model of TRALI. This finding is important for the development of safer and faster blood treatment techniques to be used in places where they are urgently needed.

## Supporting information

S1 Data Data file for Fig 1. (XLSX)

S2 Data Data file for Fig 2. (XLSX)

S3 Data Data file for Fig 3. (XLSX)

S4 Data Data file for Fig 4. (XLSX)

S5 Data Data file for Fig 5. (XLSX)
